# Outcomes of Open Versus Laparoscopic Technique in Primary Inguinal Hernia Repair: A Retrospective Study

**DOI:** 10.7759/cureus.46419

**Published:** 2023-10-03

**Authors:** Mohammed Alharthi, Alwa I Almontashri, Raghad H Alsharif, Sarah F Mozahim, Lujain K Alyazidi, Mohammed Ghunaim, Murad Aljiffry

**Affiliations:** 1 Surgery, King Abdulaziz University Faculty of Medicine, Jeddah, SAU; 2 General Surgery, King Abdulaziz University Faculty of Medicine, Jeddah, SAU; 3 Otolaryngology - Head and Neck Surgery, King Abdulaziz University Faculty of Medicine, Jeddah, SAU; 4 Anesthesia, King Abdulaziz University Faculty of Medicine, Jeddah, SAU

**Keywords:** laparoscopic inguinal hernia, open inguinal hernia, primary inguinal hernia, hernia repair outcomes, laparoscopic vs open, inguinal hernia repair

## Abstract

Inguinal hernia repair is one of the most common surgical procedures worldwide. In clinical practice, there are two different routes to repair inguinal hernias: laparoscopic mesh repair and open. Reducing the hernia and preventing recurrence remains the mainstay treatment option of both procedures. This study aims to compare postoperative outcomes and recurrence rates for patients who had primary, non-recurrent, laparoscopic, or open hernia repair in a single tertiary hospital. A retrospective cohort study was done on 468 patients. The study was conducted at King Abdulaziz University Hospital (KAUH) between 2013 and 2022. The distribution of our study population was divided into open hernia repair 378 participants (80.8%) while the rest did laparoscopic hernia repair 90 (19.2%). Operation duration in minutes was 107.158 ± 41.402 in the open hernia repair group and was noted to be significantly higher in the laparoscopic hernia repair group, with 142.811 ± 52.102 minutes p-value (0.000). The hospital length of stay was shown to be shorter in laparoscopic hernia repair (1.58 ±1.27) compared to open hernia repair (2.05±5.33). The most common postoperative complication was scrotal swelling, commonly associated with laparoscopic (5.55%) compared to 2.11% in open hernia repair. Open repair showed a risk of scrotal hematoma with a percentage of 0.52% compared to 0% in the laparoscopic method with a p-value (0.033). Hernia recurrence was non-related with any specific group, although noted to be higher in the laparoscopic group (7.77%), while in the open group (3.4%) with a p-value (0.081). The study conducted showed no alarming percentages for recurrence in either technique, open or laparoscopic, yet the open approach had a better outcome when it comes to scrotal pain and swelling post-operatively, chronic groin pain, and readmission rate as compared to laparoscopic technique, despite having a longer hospital stay. Future larger studies should be conducted to provide equal population inclusivity.

## Introduction

Inguinal hernias are one of the most common pathological conditions presented to general surgeons [1]. The lifetime prevalence of inguinal hernia is (3%-6%) in women and more prevalent in men, with a percentage range between 27% and 43% [2]. The risk increases with age, and the frequency of hernia repair escalates from 0.25% at the age of 18 to 4.2% at the age of 75-80 [3]. Family history of groin hernia, chronic obstructive pulmonary disease, smoking, high body mass index, collagen disorders, increased intra-abdominal pressure, patent processes vaginalis, previous appendectomy, and peritoneal dialysis are among the risk factors for inguinal hernia [4]. Although inguinal hernia typically presents as an asymptomatic bulge in the groin, some patients occasionally present with groin pain that worsens by the end of the day, increasing the size of the bulge, or experiencing a dragging sensation [5].

The classic herniorrhaphy techniques for inguinal hernia repair gave way to tension-free mesh repairs and, eventually, laparoscopic procedures. Ger et al. performed the first laparoscopic inguinal hernia repair procedure in 1988, and it has been demonstrated to be a successful method that offers the advantages of minimally invasive surgery. However, open or laparoscopic repair is still being debated as the best method [6]. In clinical practice, there are two different routes to repair inguinal hernias: laparoscopic mesh repair and open. Either approach can be performed by a general surgeon to manage an inguinal hernia and can vary according to various factors, such as patient characteristics, operating time, and the likelihood of complications [3]. Reducing the hernia and preventing recurrence remains the mainstay treatment goal of both procedures. Common side effects associated with hernia repair surgery are post-operative pain, prolonged hospital stay, and recurrence [3].

In many countries all over the world, hernia repair has become a day-care surgery. Patients are usually concerned about postoperative pain and prolonged hospital stay. However, surgeons performing laparoscopic hernia repair reported a decrease in hospital stay and post-operative complications [2,3].

Many scientific papers have argued about each technique's outcomes, including long- and short-term complications. However, additional research is required to illustrate the outcomes for a long period involving all the affective factors. Moreover, no studies have been done in the same field at the national level. The aim of our study is to compare postoperative complications, and the hospital stay of patients who had primary or non-recurrent, laparoscopic, or open hernia repair in King Abdulaziz University Hospital (KAUH).

## Materials and methods

Study setting

This paper is a retrospective cohort study conducted at KAUH, a tertiary center in Jeddah, Saudi Arabia. it was conducted in the Department of Surgery. The data were collected by the authors using the hospital health informatics system from the period of 2013-2022 as this time frame was selected based on the data availability. Ethical approval was obtained from the Institutional Review Board (IRB) of King Abdelaziz University (KAU) Hospital Committee, Faculty of Medicine (Reference No. 237-22).

Inclusion and exclusion criteria

All patients admitted for Inguinal hernia repair in KAUH from January 2013 to June 2022 were included. However, patients who were less than 18 years old, patients who had other types of hernia repair (e.g., ventral hernia repair), and patients who were admitted for recurrent inguinal hernia repair all were excluded from this study.

Data collection

Medical records of 468 patients were reviewed by the authors who ensured the accuracy and privacy of the data obtained including patient characteristics (age, gender, smoking status, presenting symptoms), American Society of Anaesthesiology (ASA), body mass index (BMI), past medical history (e.g., hypertension, diabetes, chronic kidney diseases), past surgical history (abdominal and non-abdominal surgeries) and risk factors (constipation, steroid consumption, strenuous activities, chronic cough, obesity).

Operative details included the operation approach (open or laparoscopic), type of operation (elective vs. emergency), duration, fixation type, and estimated blood loss. The postoperative course included postoperative disposition e.g. Intensive Care Unit (ICU), surgical site infection, surgical site hematoma, seroma, scrotal swelling, scrotal pain, scrotal hematoma, wound dehiscence, numbness, length of hospital stays, surgical reintervention, readmission, and hernia recurrence.

Data entry and data analysis

Microsoft Excel 2020 was the program used for data entry. The data analysis was performed using version 26 of IBM SPSS (IBM Corp., Armonk, NY). In describing the research, continuous variables, central tendency, and standard deviation were calculated. For categorical variables, numbers and percentages were the methods used. An independent T-test and Chi-square test were used for the evaluation of bivariate variables. The result is considered significant when the p-value is less than 0.05.

## Results

Data were obtained for analysis between 2013 and 2022. A total of 468 patients were included in the study, based on our inclusion criteria. Population mean age was older in open hernia repair 51.634 ± 18.554 versus 46.366 ± 15.00 in laparoscopic repair, which represents significance with p-value (0.010). The majority of our study participants underwent open hernia repair 378 (80.8%) while the rest did laparoscopic hernia repair 90 (19.2%). In the predominantly male sample of 442 patients, 359 (76.6%) underwent open repair, while the rest were repaired laparoscopically.

The majority of our patients belonged to ASA I 193 (50.9%) in open hernia repair, while the other group had a smaller frequency distribution, as shown in Table 1. According to our data, smoking was higher in the patients who underwent open hernia repair including 84 patients compared to the 28 who underwent laparoscopic hernia repair, with no statistical difference.

**Table 1:** Patient characteristics *Chi-square significant p-value were defined if less than 0.05.

	Total count N (%)	Open 378 (80.8%)	Laparoscopic 90 (19.2%)	P-value
Quantitative Mean ±SD				
Age at presentation	50.62± 18.032	51.634 ± 18.554	46.366 ± 15.002	0.010*
Body Mass Index (BMI)	25.807±4.810	25.933 ± 4.371	26.145 ± 4.321	0.468
Qualitative N (%)				
Gender				
Female	26 (5.6%)	19 (4.1%)	7 (1.5%)	0.306
Male	442 (94.4%)	359 (76.6%)	83 (17.7%)	
Smoking	112 (23.9%)	84	28	0.185
American Society of Anaesthesiology (ASA) score				
Class 1	242 (51.6%)	193 (50.9%)	49 (54.4%)	0.337
Class 2	195 (41.6%)	157 (41.4%)	38 (42.2%)	
Class 3	32 (6.8%)	29 (7.7%)	3 (3.3%)	
Past medical history:				
Hypertension	134 (28.6%)	115 (31.4%)	19 (21.1%)	0.056
Diabetes mellitus	80 (17.1%)	67 (18.3%)	13 (14.4%)	0.158
Chronic obstructive pulmonary disease	4 (0.9%)	3 (1.3%)	1 (1.7%)	0.002*
Chronic Kidney disease	7 (1.5%)	7 (1.9%)	-	0.168
Constipation	45 (9.6%)	39 (18.8%)	6 (10.9%)	0.329
Steroid use	18 (3.8%)	14 (3.8%)	4 (4.4%)	0.196
Obesity	62 (13.2%)	48 (12.7%)	14 (15.6%)	0.666
Chronic cough	40 (8.5%)	36 (9.5%)	4 (4.4%)	0.296
Strenuous activity	57 (12.2%)	43 (11.4%)	14 (15.6%)	0.517
Past surgical history:				
Previous abdominal surgeries	111 (23.7%)	87(23%)	24(26.6%)	0.083

The results showed that hypertension was present in a large segment of the open hernia repair group with 115 patients (31.4%), and a smaller number in the laparoscopic group with 19 patients (21.1%). Diabetes was more commonly seen in open surgery group with 18.3% vs. 14.4% in the laparoscopic group, as well as COPD, which was significant with open and repair p-value (0.002). Other risk factors are listed in Table 1.

Table 2 shows that from a total of 468 patients, the patients who operated for an elective open hernia repair were 336 patients (71.8%) compared to the patients who underwent an elective laparoscopic hernia repair with 83 patients (17.7%). However, of the patients who required emergency repair, those in the open surgery group were 42 patients (9.0%), compared to seven patients (1.5%) in the laparoscopic repair group.

**Table 2:** Operation details *Chi-square significant p-value were defined if less than 0.05.

	Total count N (%)	Open 378 (80.8%)	Laparoscopic 90 (19.2%)		P -value
Qualitative N (%)					
Type of operation:					
Elective	419 (89.5%)	336 (71.8%)	83 (17.7%)		0.353
Emergency	49 (10.5%)	42 (9.0%)	7 (1.5%)		
Fixation:					
No fixation	3 (0.6%)	3 (0.79%)	-		
Suture	287 (61.2%)	271 (71.69%)	16(17.7%)		
Tacker	52 (11.1%)	1(0.26%)	51(56.6%)		
Quantitative Mean ±SD					
Estimated blood loss	Minimal				
Duration in minutes	114.01 ± 45.815	107.158 ± 41.402		142.811 ± 52.102	*0.000

Duration of the operation in minutes was 107.158 ± 41.402 in the open hernia repair group and it was a longer duration 142.811 ± 52.102 in the laparoscopic hernia repair group with p-value (0.000). The type of fixation was by suture in 271 patients (71.69%) found in the open hernia repair group. Tacker was used in 51 patients (56.6%) in the laparoscopic hernia repair group.

Hospital stay was shorter in the laparoscopic group than in the open repair group (1.58 ± 1.27 versus 2.05 ± 5.33, p = 0.362). Scrotal swelling was significantly the most common postoperative complication, found in 13 patients among both groups, most commonly with laparoscopic cases (open 2.11% versus laparoscopic 5.55%, p = 0.015). The open technique was associated with a higher risk of scrotal hematoma (open 0.52% versus laparoscopic 0%, p = 0.033). Scrotal pain was more commonly noted among the laparoscopic group (open 0.26% versus laparoscopic 1.11%, p = 0.024). Surgical site infection was recorded in four patients, three of whom were in the open repair group (0.7%), but the difference lacks statistical significance.

**Figure 1:**
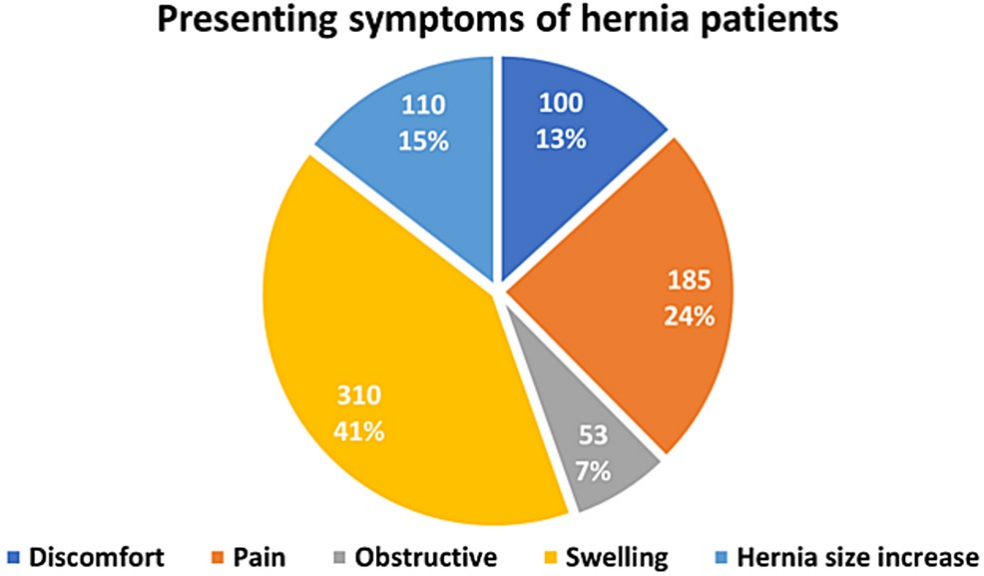
Hernia patients presentation

The laparoscopic technique was associated with postoperative chronic groin pain in 1.11% of the laparoscopic group, whereas the open group had only 0.26% with a p of 0.036. Hernia recurrence was noted to be higher in the laparoscopic group (7.77%), while in the open group, the recurrence rate was p = 0.081 (3.4%). The laparoscopic approach was associated with a significant higher hospital readmission than the open technique (laparoscopic 2.22% vs. open 0.52%, p = 0.029). Table 3 shows these results. Figure 1 shows the presenting symptoms among hernia patients. Swelling was the most common presenting symptoms and was seen in 301 patients (41%) followed by pain in 185 patients (24%).

**Table 3:** Operation method versus postoperative morbidity *ICU= Intensive Care Unit *Chi square significant p-value were defined if less than 0.05.

Morbidity	Total count N (%)	Open 378 (80.8%)	Laparoscopic 90 (19.2%)	P -value
Qualitative N (%)				
Length of hospital stay	1.96 ± 4.823	2.05 ± 5.33	1.58 ± 1.27	0.362
Surgical site infection	4 (0.9%)	3(0.7%)	1 (1.11%)	0.135
Hematoma	6 (1.3%)	5 (1.3%)	1(1.11%)	0.063
Seroma	7 (1.5%)	5 (1.3%)	2(2.2%)	0.048*
Scrotal swelling	13 (2.8%)	8 (2.11%)	5 (5.55%)	0.015*
Scrotal hematoma	2 (0.4%)	2 (0.52%)	-	0.033*
Numbness	6 (1.3%)	6(1.6%)	-	0.025*
Scrotal pain	2 (0.4%)	1 (0.26%)	1(1.11%)	0.024*
Surgical reintervention	1 (0.2%)	-	1(1.11%)	0.007*
Chronic groin pain	2 (0.4%)	1(0.3%)	1(1.11%)	0.036*
ICU admission*	6 (1.3%)	5 (1.5%)	1(1.11%)	0.093
Hernia recurrence	20 (4.3%)	13(3.4%)	7(7.77%)	0.081
Hospital readmission	4 (0.9%)	2(0.52%)	2(2.22%)	0.029*

Total hernia recurrence was 20 cases, 13 (3.4%) of which were through the open approach, whereas the rest were laparoscopic (7.7%). A history of hypertension and steroid usage showed significant association with open hernia recurrence, whereas diabetes was found to be more associated with laparoscopic hernia recurrence, with p-value of 0.007 vs. 0.043 vs. 0.034 respectively.

The open mesh fixation approach with sutures showed significant recurrence (69.2%), compared to no fixation (7.7%), whereas in laparoscopic mesh fixation, recurrence was high with tackers (71.4%) with a p-value (0.000). This is due to the fact that the largest percentage of mesh fixation in the open approach group was done with suture fixation and in the laparoscopic group with tackers.

Post laparoscopic complication including seroma and scrotal swelling were risk factors for recurrence with a p-value (0.000 vs 0.019) respectively. Surgical site infection, surgical site hematoma and scrotal hematoma showed no association with hernia recurrence in either approach. Other causative factors are listed in Table 4.

**Table 4:** Possible precipitating factors in patients who had hernia recurrence *ICU= Intensive Care Unit. *Chi square significant p-value were defined if less than 0.05.

Factors	Open hernial recurrence 13 (3.4%)	Laparoscopic hernial recurrence 7 (7.8%)	P-value
Qualitative N (%)			
Gender			
Male	12 (92.3%)	7 (100%)	0.307
Female	1 (7.7%)	-	
Smoking	4 (30.8%)	3 (42.9%)	0.725
Past medical history			
Hypertension	4 (30.8%)	1 (14.3%)	0.007
Diabetes mellitus	4 (30.8%)	3 (42.9%)	0.034
Steroid use	2 (15.4%)	-	0.043
Obesity	3 (23.1%)	1 (14.3%)	0.754
Chronic cough	2 (15.4%)	-	0.738
Strenuous activity	2 (15.4%)	1 (14.3%)	0.655
BPH	3 (23.1%)	-	0.972
Type of operation			
Elective	(84.6%) 11	7 (100.0%)	0.354
Emergency	2 (15.4%)	-	
Fixation:			
No fixation	1 (7.7%)	-	0.000
Suture	9 (69.2%)	1 (14.3%)	
Tacker	-	5 (71.4%)	
Post operative complication			
Seroma	-	1 (14.3%)	0.000
Scrotal swelling	1 (7.7%)	2 (28.%)	0.019
Scrotal pain	-	1(14.3%)	0.000
Orchitis	-	1 (14.3%)	0.000

## Discussion

This retrospective observational study investigates the recurrence rate and outcome between two techniques of hernia repair in a primary hernia presentation. Inguinal hernias occur in about 3.8% of the general population and its repair is one of the most common operations in general surgical practice [3].

Laparoscopic hernia repair has recently gained a wild acceptance among surgeons since it offers a shorter hospital stay, fewer post-operative complications, and s quicker return to work [7]. The majority of our study population had undergone open hernia repair in comparison to only 90 cases operated laparoscopically. This could be explained by senior surgeons’ preference, slow acceptance of the new laparoscopic method, and the difficulties in adopting to different surgical approach [7,8].

The average time for an open hernia repair was 107.158 ± 41.402 minutes, while the average for a laparoscopic procedure was 142.811 ± 52.102 minutes, which is similar to previous studies where the laparoscopic repair was found to have a longer duration than the open repair [7,9-11]. This could be explained by the duration of setting the equipment and the expertise needed for performing the laparoscopic technique. However, some studies reported a similar operative time in both groups [12,13].

The incidence of early postoperative complications was lower after open repair compared to laparoscopic repair, with a significant difference between the groups. In contrast, some studies have shown that the laparoscopic approach tends to have fewer complications. Whereas no difference was documented in other studies [7,14,15]. This could be explained by the differences in surgeons’ capabilities, training, and volume exposure.

In addition, a shorter hospital stay was seen in the laparoscopic repair group with a mean of 1.58 ± 1.27 with no difference between groups. This is similar to previous studies where laparoscopic hernia repair is a one-day procedure [16-18].

Previous studies show that the recurrence rate is higher in open hernia repair [11]. Another study comparing both methods found no difference, whereas previous meta-analysis concluded that the recurrence was higher in the laparoscopic hernia repair, mainly depending on the surgeons’ skills [8,9,19]. Despite the previous studies, our study shows that the total hernia recurrence was 20 cases, with seven cases (7.8%) of the recurrence in the laparoscopic approach group with no statistical differences.

Previous multiple meta-analyses concluded that in laparoscopic repair for defects up to 3 cm, mesh without fixation does not raise the recurrence rate over mesh with fixation [20]. Moreover, various meta-analyses did not find differences in recurrence rate if glue or tucker fixation was used. Additionally, previous studies of the open procedure documented that both suture fixation and glue had the same recurrence rate [20-22]. Nevertheless, in our recent study open mesh fixation approach with suture showed significant recurrence (69.2%) with a p-value (0.000).

This article is a single institution retrospective study, the parameters of which were limited to the documentation available, and the participants given. The sample distribution was unbalanced, due to the diversities of the performing surgeons and their preferences. A prospective study could solve this study’s limitations and also assess the patients for longer follow-ups.

## Conclusions

Although there was no significant recurrence in both groups, open hernia repair carried a better outcome despite open hernia repair carried a better outcome despite having longer hospital duration Although there was no significant recurrence in both groups. We recommend that more prospective studies with a larger sample size, more balanced between open and laparoscopic hernia repair be performed to understand these differences between the two techniques. We hope our study will let the surgeon take into consideration the outcome between both types of hernia repair and implicate that to a patient personalized plan.
